# Distinguishing Frontotemporal Lobar Degeneration Tau from TDP‐43 using white matter microstructure

**DOI:** 10.1002/alz.092085

**Published:** 2025-01-09

**Authors:** Hamsanandini Radhakrishnan, Philip A. Cook, Jeffrey S Phillips, Christopher A Olm, Christopher D. Brown, James C. Gee, Eddie B Lee, Vivianna M Van Deerlin, David J Irwin, Lauren Massimo, Corey T McMillan

**Affiliations:** ^1^ University of Pennsylvania, Philadelphia, PA USA; ^2^ Department of Radiology, University of Pennsylvania, Philadelphia, PA USA; ^3^ Department of Neurology, University of Pennsylvania, Philadelphia, PA USA; ^4^ Penn Image Computing and Science Laboratory, Philadelphia, PA USA; ^5^ University of Pennsylvania, School of Nursing, Philadelphia, PA USA; ^6^ Penn Frontotemporal Degeneration Center, Department of Neurology, Perelman School of Medicine, University of Pennsylvania, Philadelphia, PA USA

## Abstract

**Background:**

Frontotemporal lobar degeneration (FTLD) is defined neuropathologically by misfolded tau (FTLD‐tau) or TAR DNA‐binding protein of 43 kDa (FTLD‐TDP). However, we lack biomarkers that can distinguish them in vivo which is a major barrier to effective disease‐modifying treatment trials. Based on neuropathological evidence of distinct patterns of cellular degeneration, more prominent in white matter (WM) for FTLD‐tau relative to FTLD‐TDP, we hypothesized that diffusion MRI (dMRI) measures of white matter microstructure would help dissociate FTLD‐tau and FTLD‐TDP during life.

**Method:**

We evaluated dMRI for 157 individuals with FTLD‐Tau (n=66, autopsy‐confirmed sporadic=51, MAPT=15) and FTLD‐TDP (n=91, autopsy‐confirmed sporadic=25, GRN=19, C9orf72=47) defined by a pathogenic genetic mutation or autopsy confirmation. Images were preprocessed using QSIprep and reconstructed using Generalized Q‐sampling Imaging. Tensor‐based scalar maps of fractional anisotropy (FA) and mean diffusivity (MD) were then calculated. Deterministic tractography was run and white matter bundles of pathogenic interest based on literature were segmented with DSIstudio using template‐based tracking.

**Result:**

Overall, FTLD‐Tau individuals showed worse white matter integrity compared to those with FTLD‐TDP with lower FA in the superior longitudinal fasciculus (SLF), uncinate fasciculus and fronto‐parietal cingulum; and higher MD in the uncinate fasciculus. Even within sporadic autopsy‐confirmed individuals, FTLD‐Tau subtypes had significantly lower FA in the SLF, fronto‐parietal cingulum, and left corticostriatal tract relative to FTLD‐TDP.

**Conclusion:**

Our results demonstrate that white matter microstructure can distinguish between FTLD‐Tau and FTLD‐TDP pathologies in a tract‐specific manner with greater evidence of white matter degeneration in disease‐associated regions in FTLD‐Tau. Future work examining longitudinal associations with disease progression and white matter microstructure could aid in non‐invasive differentiation of these subtypes enabling more sensitive treatment monitoring.

